# Supplementary material for: Cannibalism makes invasive comb jelly, Mnemiopsis leidyi, resilient to unfavourable conditions
Source: Commun Biol. 2020 May 7;3:212. doi: 10.1038/s42003-020-0940-2 (PMC7205609; doi:10.1038/s42003-020-0940-2)
Supplement: Supplementary file 1 — Reporting Summary [file 42003_2020_940_MOESM1_ESM.pdf]

nature research

Corresponding author(s): Thomas Larsen

Last updated by author(s): Mar 18, 2020

Reporting Summary

Nature Research wishes to improve the reproducibility of the work that we publish. This form provides structure for consistency and transparency in reporting. For further information on Nature Research policies, see Authors & Referees and the Editorial Policy Checklist.

Statistics

For all statistical analyses, confirm that the following items are present in the figure legend, table legend, main text, or Methods section.

n/a	Confirmed	
☐	☒	The exact sample size (*n*) for each experimental group/condition, given as a discrete number and unit of measurement
☐	☒	A statement on whether measurements were taken from distinct samples or whether the same sample was measured repeatedly
☐	☒	The statistical test(s) used AND whether they are one- or two-sided<br>*Only common tests should be described solely by name; describe more complex techniques in the Methods section.*
☐	☒	A description of all covariates tested
☐	☒	A description of any assumptions or corrections, such as tests of normality and adjustment for multiple comparisons
☐	☒	A full description of the statistical parameters including central tendency (e.g. means) or other basic estimates (e.g. regression coefficient) AND variation (e.g. standard deviation) or associated estimates of uncertainty (e.g. confidence intervals)
☐	☒	For null hypothesis testing, the test statistic (e.g. *F*, *t*, *r*) with confidence intervals, effect sizes, degrees of freedom and *P* value noted<br>*Give P values as exact values whenever suitable.*
☒	☐	For Bayesian analysis, information on the choice of priors and Markov chain Monte Carlo settings
☒	☐	For hierarchical and complex designs, identification of the appropriate level for tests and full reporting of outcomes
☒	☐	Estimates of effect sizes (e.g. Cohen's *d*, Pearson's *r*), indicating how they were calculated

Our web collection on statistics for biologists contains articles on many of the points above.

Software and code

Policy information about availability of computer code

Data collection	NA
Data analysis	To partition the net effects of environmental variables on population growth and decline and their relative importance we constructed two separated structural equation models (SEM) to assess the different states of the Mnemiopsis leidyi population, e.g. growth and collapse. The first model assessed population growth and included abiotic (temperature and salinity) and biotic factors (micro and mesozooplankton). The second model assessed drivers of population collapse, which included a density-dependent factor (adult abundance), besides the above abiotic and biotic factors. Based on previous observations (Javid et al. 2009) we hypothesized that (i) M. leidyi population growth is driven by food availability, and the match with warming condition, (ii) while the population collapse may response to food depletion, temperature decline and cannibalism.<br>Data were separated into two groups, see Fig. 3, and analyzed in a framework of multigroup SEM to assess whether the relative importance of environmental effects (abiotic and biotic) vary in growth and collapse population states. In the multigroup SEM, we assessed the effects of three scenarios to model the effect of forcing variables (i) population growth driven by hydrological conditions and food availability, (ii) population collapse driven by hydrological conditions and food availability, and (iii) population collapse driven by hydrological conditions, food availability and cannibalism.<br>To analyse the data we compared models with the observed covariance matrix, using maximum likelihood and $\chi 2$ as goodness-of-fit measures. When $P<0.05$ data were considered significantly different from the model. As data from the individual groups fit the model ($P > 0.05$), we considered legitimate to perform a multigroup SEM analysis. Significance levels for individual paths between variables were set at $\alpha = 0.05$. SEMs were run in AMOS (version 21).<br>No specific code was developed for Fig.4; equations used to estimate daily ration are included in the main text. The estimates were generated in Matlab R2018, and the results were plotted with Sigmaplot v.14.

For manuscripts utilizing custom algorithms or software that are central to the research but not yet described in published literature, software must be made available to editors/reviewers. We strongly encourage code deposition in a community repository (e.g. GitHub). See the Nature Research guidelines for submitting code & software for further information.

Data

Policy information about availability of data

All manuscripts must include a data availability statement. This statement should provide the following information, where applicable:

- Accession codes, unique identifiers, or web links for publicly available datasets
- A list of figures that have associated raw data
- A description of any restrictions on data availability

The complete data set used in our study has been deposited at PANGAEA, see data repository PANGAEA PDI-17499.

Field-specific reporting

Please select the one below that is the best fit for your research. If you are not sure, read the appropriate sections before making your selection.

☐ Life sciences ☐ Behavioural & social sciences ☒ Ecological, evolutionary & environmental sciences

For a reference copy of the document with all sections, see nature.com/documents/nr-reporting-summary-flat.pdf

Life sciences study design

All studies must disclose on these points even when the disclosure is negative.

Sample size	*Describe how sample size was determined, detailing any statistical methods used to predetermine sample size OR if no sample-size calculation was performed, describe how sample sizes were chosen and provide a rationale for why these sample sizes are sufficient.*
Data exclusions	*Describe any data exclusions. If no data were excluded from the analyses, state so OR if data were excluded, describe the exclusions and the rationale behind them, indicating whether exclusion criteria were pre-established.*
Replication	*Describe the measures taken to verify the reproducibility of the experimental findings. If all attempts at replication were successful, confirm this OR if there are any findings that were not replicated or cannot be reproduced, note this and describe why.*
Randomization	*Describe how samples/organisms/participants were allocated into experimental groups. If allocation was not random, describe how covariates were controlled OR if this is not relevant to your study, explain why.*
Blinding	*Describe whether the investigators were blinded to group allocation during data collection and/or analysis. If blinding was not possible, describe why OR explain why blinding was not relevant to your study.*

Behavioural & social sciences study design

All studies must disclose on these points even when the disclosure is negative.

Study description	*Briefly describe the study type including whether data are quantitative, qualitative, or mixed-methods (e.g. qualitative cross-sectional, quantitative experimental, mixed-methods case study).*
Research sample	*State the research sample (e.g. Harvard university undergraduates, villagers in rural India) and provide relevant demographic information (e.g. age, sex) and indicate whether the sample is representative. Provide a rationale for the study sample chosen. For studies involving existing datasets, please describe the dataset and source.*
Sampling strategy	*Describe the sampling procedure (e.g. random, snowball, stratified, convenience). Describe the statistical methods that were used to predetermine sample size OR if no sample-size calculation was performed, describe how sample sizes were chosen and provide a rationale for why these sample sizes are sufficient. For qualitative data, please indicate whether data saturation was considered, and what criteria were used to decide that no further sampling was needed.*
Data collection	*Provide details about the data collection procedure, including the instruments or devices used to record the data (e.g. pen and paper, computer, eye tracker, video or audio equipment) whether anyone was present besides the participant(s) and the researcher, and whether the researcher was blind to experimental condition and/or the study hypothesis during data collection.*
Timing	*Indicate the start and stop dates of data collection. If there is a gap between collection periods, state the dates for each sample cohort.*
Data exclusions	*If no data were excluded from the analyses, state so OR if data were excluded, provide the exact number of exclusions and the rationale behind them, indicating whether exclusion criteria were pre-established.*
Non-participation	*State how many participants dropped out/declined participation and the reason(s) given OR provide response rate OR state that no participants dropped out/declined participation.*
Randomization	*If participants were not allocated into experimental groups, state so OR describe how participants were allocated to groups, and if allocation was not random, describe how covariates were controlled.*

Ecological, evolutionary & environmental sciences study design

All studies must disclose on these points even when the disclosure is negative.

Study description	We used an approach based on fieldwork, experimental analysis and modeling. Field data were used to assess partitioning net effects of environmental variables, and estimating their relative importance on population growth and decline using structural equation modelling (SEM). Field data were also used to quantifying the daily ration of adults, as defined by the ingestion rate per capita.<br><br>We conducted a laboratory culture experiment to test whether adult comb jellies, Mnemiopsis leidyi, consume their larvae. To do so, we incubated one adult M. leidyi together with ten 15N labeled M. leidyi larvae for a period of 36 h in 2-liter jars at GEOMAR. In our control treatment, we incubated one adult M. leidyi together with ten non-labeled M. leidyi larvae under similar experimental conditions. From the mass and 15N values of both larvae and adult M. leidyi, we performed mass-balance calculations to estimate the extent adults assimilated larvae. The M. leidyi specimens used in the feeding trial were obtained from a permanent culture at GEOMAR that originated from wild specimens collected in Kiel Fjord. The food source of M. leidyi larvae was viable Acartia tonsa obtained from permanent cultures at GEOMAR. The copepods were reared on cryptophyte algae. The 15N-labeled algae were obtained by culturing one batch with 15N-(NH4)2SO4 in the culture medium. Both the control and experimental feeding trials were conducted in triplicate. See Materials and Methods for further details on algal culturing and the feeding trials.
Research sample	A high frequency field survey was performed in the Kiel Fjord, south-western Baltic Sea, to capture the daily dynamics prior, during and after M. leidyi population bloom. Zooplankton samples were taken daily from the bottom (6 m) to the surface and M. Leidyi individuals counted and measured alive immediately after sampling because M. leidyi disintegrates in standard fixation solutions. Microzooplankton were counted in the laboratory using a convert microscope, while mesozooplankton organisms were counted under a dissecting microscope.
Sampling strategy	Net sampling was used to collect individuals for both feeding experiment and field data. Individuals that were used for obtaining visual evidence of cannibalism were immediately selected and acclimated into our experimental jars. Field samples were counted and sized right after sampling as comb jellies cannot be preserved due to their fragile nature.
Data collection	Field samples and data were collected from Kiel Fjord in the Western Baltic Sea, laboratory and environmental data from GEOMAR in Kiel, and isotope analysis was performed by the Centre for Stable Isotope Research and Analysis at University of Göttingen.
Timing and spatial scale	The measurements on metabolic rates was conducted in January 2007, the high resolution field sampling was conducted in throughout 2008, the feeding trial in September 2016, and the visual evidence of cannibalism among field collected adults was obtained in September 2008. All field samples were obtained from location 54° 19´48´´N, 10°9´1´´E (see map in Fig. 1).
Data exclusions	NA
Reproducibility	Plankton sampling was performed at the GEOMAR deck between 10.00 and 11.00 h from Mondays to Saturdays in the period of August 12 to October 21, 2008. During the same period and from the same station, mesozooplankton were sampled in weekly intervals. For each sampling occasion, we sampled with a WP2 net making three vertical hauls from the bottom (6 m) to the surface. Both the control and experimental feeding trials were conducted in triplicate.<br>For both field and laboratory experiments, we used all the collected data in our data analysis.
Randomization	For the feeding trial, specimens for the control and feeding trials were selected randomly. NA for the field sampling
Blinding	It was not relevant to perform blinded trials for the feeding trials because handling, sampling and analyses were identical in both the control and treatment trials.

Did the study involve field work? ☒ Yes ☐ No

Field work, collection and transport

Field conditions	Hydrographic and climatic data such as wind direction, wind speed, and water level were obtained from meteorological station at the roof of the institute.
Location	At the GEOMAR deck (54° 19´48´´N, 10°9´1´´E, see map in Fig. 1)
Access and import/export	Samples were processed at GEOMAR immediately after sampling, and the isotope analysis was performed in Germany.
Disturbance	NA

Reporting for specific materials, systems and methods

We require information from authors about some types of materials, experimental systems and methods used in many studies. Here, indicate whether each material, system or method listed is relevant to your study. If you are not sure if a list item applies to your research, read the appropriate section before selecting a response.

Materials & experimental systems

n/a	Involved in the study
☒	☐ Antibodies
☒	☐ Eukaryotic cell lines
☒	☐ Palaeontology
☐	☒ Animals and other organisms
☒	☐ Human research participants
☒	☐ Clinical data

Methods

n/a	Involved in the study
☒	☐ ChIP-seq
☒	☐ Flow cytometry
☒	☐ MRI-based neuroimaging

Antibodies

Antibodies used	NA
Validation	NA

Eukaryotic cell lines

Policy information about cell lines

Cell line source(s)	NA
Authentication	NA
Mycoplasma contamination	NA
Commonly misidentified lines (See ICLAC register)	NA

Palaeontology

Specimen provenance	NA
Specimen deposition	NA
Dating methods	NA

☐ Tick this box to confirm that the raw and calibrated dates are available in the paper or in Supplementary Information.

Animals and other organisms

Policy information about studies involving animals; ARRIVE guidelines recommended for reporting animal research

Laboratory animals	M. leidyi are non-vertebrate animals and do not fall under the ethical guidelines of 'laboratory animals'.
Wild animals	Invasive comb jelly, M. leidyi.
Field-collected samples	Plankton sampling was performed at the GEOMAR deck (54° 19´48´´N, 10°9´1´´E, see map in Fig. 1) between 10.00 and 11.00 h from Mondays to Saturdays in the period of August 12 to October 21, 2008. Samples of M. leidyi were taken with a WP2 net (0.8 m net opening, 500 µm mesh size) making vertical hauls from the bottom (6 m) to the surface. Individuals were counted and measured alive immediately after sampling since M. leidyi disintegrate in standard fixation solutions. Total length was measured to the nearest 0.1 mm on individuals with closed lobes. Mesozooplankton were sampled at the same station in weekly intervals by using a plankton net (0.6 m diameter opening, 200 µm mesh size) from integrated vertical tows of 6 m depth to the surface. Samples were preserved in 5% buffered formaldehyde-seawater mixture for later quantification. All mesozooplankton specimens in the samples were identified at least to genus level under a dissecting microscope (see data repository PANGAEA PDI-17499 and Paulsen, M. et al., Ices J Mar Sci 71, 991-1000, 2013). Water samples (250 ml) were taken from the mid-depth on a weekly basis and were preserved using Acid-Lugol for later counts of microzooplankton. Microzooplankton species composition was determined at least to the genus level using a convert microscope. Temperature and salinity were measured at a one-meter interval along the whole water column at each sampling day.
Ethics oversight	No ethical statement is needed.

Note that full information on the approval of the study protocol must also be provided in the manuscript.

Human research participants

Policy information about studies involving human research participants

Population characteristics	NA
Recruitment	NA
Ethics oversight	NA

Note that full information on the approval of the study protocol must also be provided in the manuscript.

Clinical data

Policy information about clinical studies

All manuscripts should comply with the ICMJE guidelines for publication of clinical research and a completed CONSORT checklist must be included with all submissions.

Clinical trial registration	NA
Study protocol	NA
Data collection	NA
Outcomes	NA

ChIP-seq

Data deposition

☐ Confirm that both raw and final processed data have been deposited in a public database such as GEO.

☐ Confirm that you have deposited or provided access to graph files (e.g. BED files) for the called peaks.

Data access links<br>*May remain private before publication.*	NA
Files in database submission	NA
Genome browser session<br>(e.g. UCSC)	NA

Methodology

Replicates	NA
Sequencing depth	NA
Antibodies	NA
Peak calling parameters	NA
Data quality	NA
Software	NA

Flow Cytometry

Plots

Confirm that:

☐ The axis labels state the marker and fluorochrome used (e.g. CD4-FITC).

☐ The axis scales are clearly visible. Include numbers along axes only for bottom left plot of group (a 'group' is an analysis of identical markers).

☐ All plots are contour plots with outliers or pseudocolor plots.

☐ A numerical value for number of cells or percentage (with statistics) is provided.

Methodology

Sample preparation	NA
Instrument	NA
Software	NA
Cell population abundance	NA
Gating strategy	NA

☐ Tick this box to confirm that a figure exemplifying the gating strategy is provided in the Supplementary Information.

Magnetic resonance imaging

Experimental design

Design type	NA
Design specifications	NA
Behavioral performance measures	NA

Acquisition

Imaging type(s)	NA
Field strength	NA
Sequence & imaging parameters	NA
Area of acquisition	NA

Diffusion MRI ☐ Used ☐ Not used

Preprocessing

Preprocessing software	NA
Normalization	NA
Normalization template	NA
Noise and artifact removal	NA
Volume censoring	NA

Statistical modeling & inference

Model type and settings	NA
Effect(s) tested	NA

Specify type of analysis: ☐ Whole brain ☐ ROI-based ☐ Both

Statistic type for inference (See Eklund et al. 2016)	NA
Correction	NA

Models & analysis

n/a	Involved in the study
☒	☐ Functional and/or effective connectivity
☒	☐ Graph analysis
☒	☐ Multivariate modeling or predictive analysis
