# Supplementary material for: Cannibalism makes invasive comb jelly, Mnemiopsis leidyi, resilient to unfavourable conditions
Source: Commun Biol. 2020 May 7;3:212. doi: 10.1038/s42003-020-0940-2 (PMC7205609; doi:10.1038/s42003-020-0940-2)
Supplement: Supplementary file 2 — Supplementary Information [file 42003_2020_940_MOESM2_ESM.pdf]

Supplementary Figure 1: Photographic evidence of cannibalistic behaviour

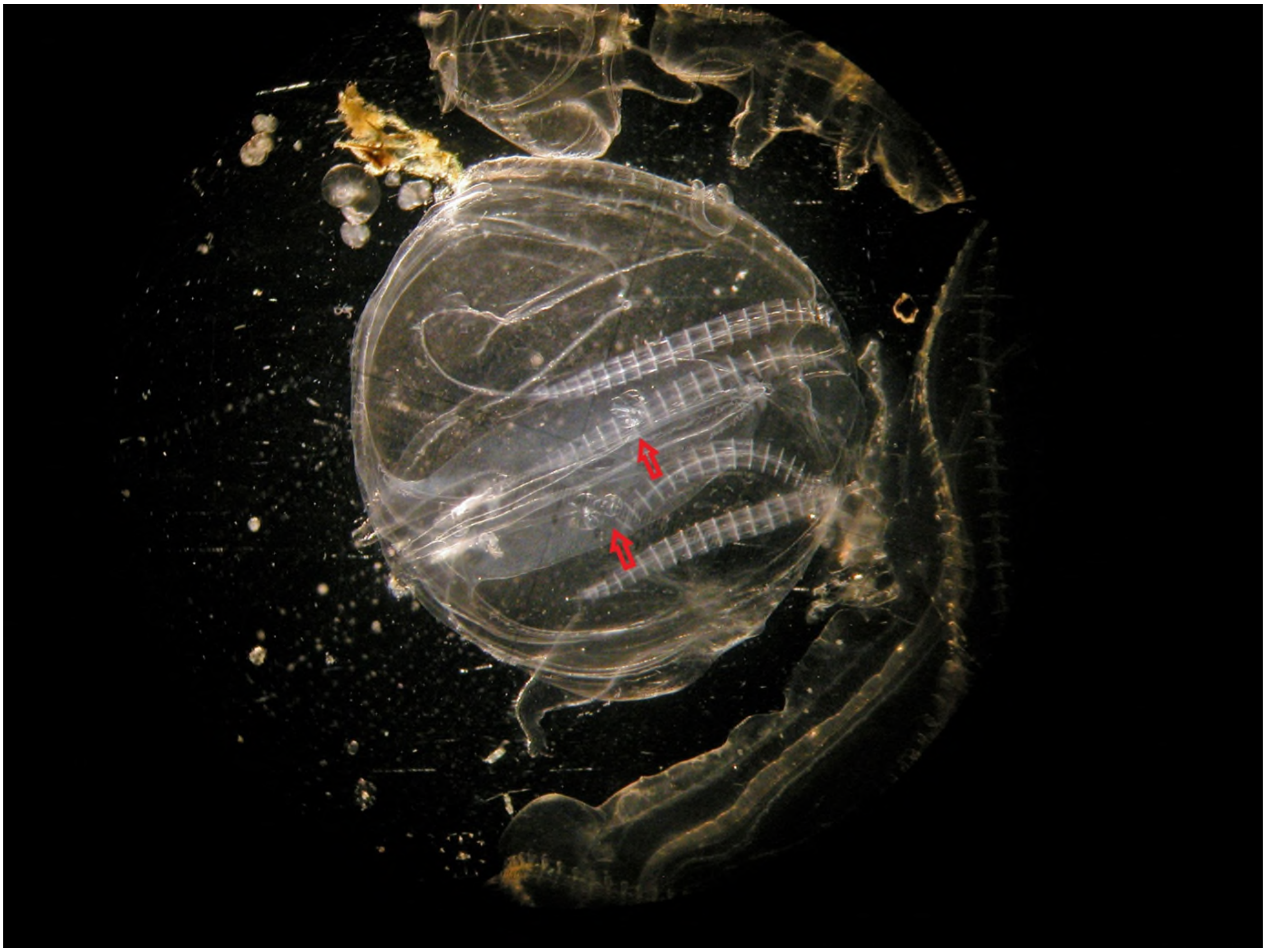

Supplementary Figure 1. *Mnemiopsis leidyi* larvae (next to red arrows) located within the auricles of an adult. The specimens were captured September 29, 2008 in Kiel Fjord during population sampling of adult *M. leidyi* and photographed approximately 30 min later in the laboratory of GEOMAR.

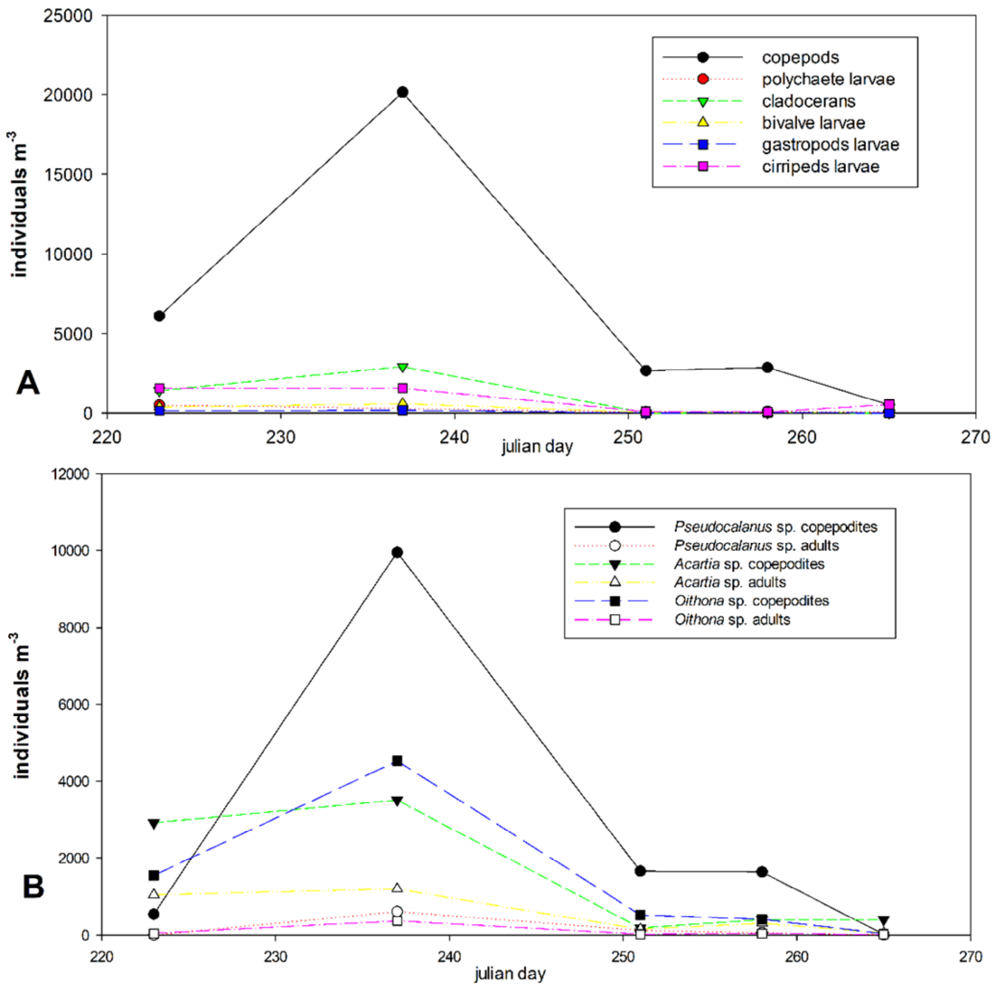

Supplementary Figure 2. A) The mesozooplankton composition during late summer 2008 in Kiel Fjord shows that the community was dominated by copepods. B) The copepod assemblages during the same period shows that *Pseudocalanus* sp. was the most abundant group during the bloom followed by copepodites of *Oithona* sp. and *Acartia* sp.

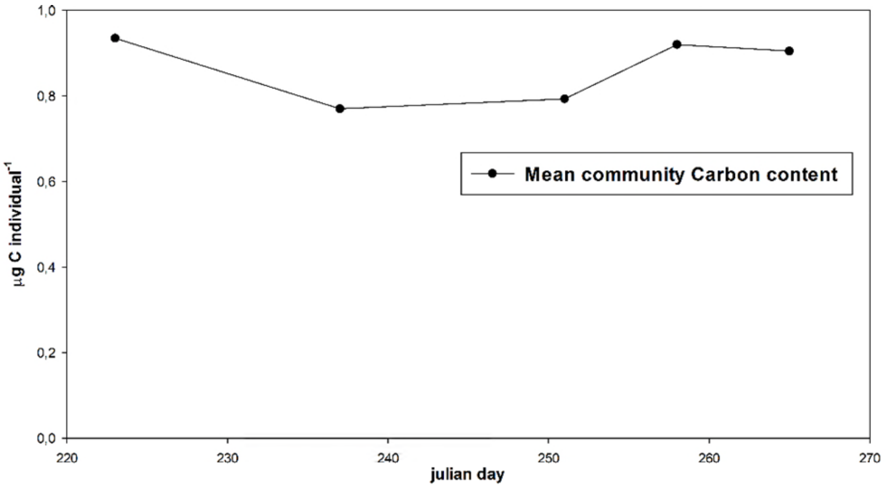

Supplementary Figure 3. The mean community carbon content per individual copepod based on the carbon contents in Supplementary Table 1 and the assemblages in Supplementary Figure 2B. We used an averaged value of 0.9 µg C indiv^{-1} to calculate daily ration rates.

Supplementary Table 1. Individual carbon contents of copepods.

**Group**	**Carbon content (μg C indiv^{-1})**	**Reference**
Acartia sp. copepodites	0.8	a
Acartia sp. adults	2.5	b
Oithona sp. copepodites	0.17	b
Oithona sp. adults	0.5	b

a. Jones, R.H., Flynn, K.J. & Anderson, T.R. Effect of food quality on carbon and nitrogen growth efficiency in the copepod *Acartia tonsa*. Marine Ecology Progress Series 235, 147-156 (2002).
b. Granhag, L., Møller, L.F. & Hansson, L.J. Size-specific clearance rates of the ctenophore *Mnemiopsis leidyi* based on in situ gut content analyses. Journal of Plankton Research 33, 1043-1052 (2011).
